# Identification of the putative gene encoding the RNA subunit of telomerase in *Malassezia* clade species through comparative genomic analysis

**DOI:** 10.1093/g3journal/jkaf185

**Published:** 2025-08-19

**Authors:** Juan Antonio Sanpedro-Luna, Patricia Sanchez-Alonso

**Affiliations:** Centro de Investigaciones en Ciencias Microbiológicas, Instituto de Ciencias, Benemérita Universidad Autónoma de Puebla, Av. San Claudio y 24 Sur Edif, IC11 PB. P.O. 72570, Puebla, México; Centro de Investigaciones en Ciencias Microbiológicas, Instituto de Ciencias, Benemérita Universidad Autónoma de Puebla, Av. San Claudio y 24 Sur Edif, IC11 PB. P.O. 72570, Puebla, México

**Keywords:** *Malassezia* spp, telomerase RNA subunit, comparative genomics

## Abstract

Maintaining telomere length and genomic stability in eukaryotic cells is dependent on telomerase, a ribonucleoprotein with reverse transcriptase activity that counteracts the end replication problem. Its core components include telomerase reverse transcriptase (TERT) and telomerase RNA (TER). This study focused on the identification of the putative *MgTER* gene, which encodes the telomerase RNA subunit, in *Malassezia globosa* through comparative genomics. Unlike the corresponding genes in other yeasts and filamentous fungi, this gene is compact and uniform in length, resembling similar genes in vertebrates, while retaining characteristic elements that are crucial for TER and snRNA biogenesis and activity. These elements include the template domain, pseudoknot domain, CR4/5 domain, and a putative Sm-binding site. Furthermore, transcriptomic analysis confirmed the transcriptional activity of the putative *MgTER* gene, reinforcing its potential functional significance within this organism. These results establish a robust basis for future experimental investigations to verify the functionality and regulatory mechanisms of this gene, particularly its role in telomere maintenance and broader cellular processes.

## Introduction

Telomerase is a ribonucleoprotein with reverse transcriptase activity that catalyzes the de novo synthesis of telomeric repeats at the linear ends of eukaryotic chromosomes (Greider and Blackburn 1989). Telomerase activity addresses the end replication problem (Olovnikov 1973; Watson 1972) by elongating telomeres. The maintenance of telomere length by telomerase allows telomeres to act as protective caps on chromosome ends and prevent DNA repair machinery from recognizing these ends as double-strand breaks. Therefore, the activity of telomerase is critical and contributes to the maintenance of genomic stability by preventing the erosion of chromosomal end sequences and fusion-bridge-breakage cycles (Ducray et al. 1999; Hackett et al. 2001). The core components that form the catalytic core of the complex required for the restoration of catalytic activity in vitro are the protein subunit telomerase reverse transcriptase (TERT), which functions as a reverse transcriptase, and the subunit telomerase RNA (TER), which harbors the template motif for reverse transcription and acts as a scaffold for TERT and components involved in complex activity (Nguyen et al. 2018).

In addition to its telomeric function, TER may have extratelomeric functions in different organisms, including the regulation of key biological processes. It has been reported that this subunit plays an antiapoptotic role in response to cellular stress (Gazzaniga and Blackburn 2014; Rubtsova et al. 2018), participates in the DNA damage response (Kedde et al. 2006; Ting et al. 2009), and regulates gene expression and cell differentiation (Alcaraz-Perez et al. 2014; Li et al. 2005); moreover, in dimorphic fungi, TER has been shown to participate in regulating genes that are involved in the life cycle and pathogenic development (Logeswaran et al. 2022; Sanpedro-Luna et al. 2023). These findings show that the telomerase RNA subunit plays broader roles in cellular biology beyond its function in telomere stability, making the identification of genes related to telomeres and telomerase in various organisms an area of interest in biological research.

TER exhibits high divergence across organisms, even among closely related species (Dandjinou et al. 2004; Zappulla and Cech 2004). They vary in sequence composition and length, displaying considerable structural plasticity (Zappulla 2020; Zappulla and Cech 2004), which presents major challenges for their identification through bioinformatics approaches (Waldl et al. 2018). To date, fungal TERs have been identified and extensively characterized in the phylum Ascomycota (Gunisova et al. 2009; Kachouri-Lafond et al. 2009; Singer and Gottschling 1994), whereas their identification in certain Ustilaginomycetes species has only recently been reported (Logeswaran et al. 2022; Sanpedro-Luna et al. 2023).

In contrast to species within the subphylum Saccharomycotina, which possess unusually long and heterogeneous telomeric repeats (Sun et al. 2023), members of the phylum Basidiomycota predominantly harbor vertebrate-type TTAGGG repeats (Edman 1992; Guzman and Sanchez 1994; Perez et al. 2009). This suggests that their telomeric machinery interacts with DNA substrates with comparable properties (Yu et al. 2020, 2013).

With the recent advances in the genomic characterization of the genus *Malassezia*, it has been revealed that the telomere repeat motif TTAGGG from vertebrates, or subtle variations of it, is shared with at least 18 species within this clade (Coelho et al. 2023). Furthermore, the genome annotations of the studied group include a unique open reading frame that conserves the distinctive domains of telomerase catalytic subunits found in humans, some plants, and several filamentous fungi (Coelho et al. 2023; Xu et al. 2007), backing the idea that, as in most eukaryotic groups, telomere maintenance in *Malassezia* is mediated by the telomerase pathway; yet, the study of telomerase components and their functions has recently begun to be explored in this fungus.

The genus *Malassezia* belongs to the class Malasseziomycetes and is classified within the division Basidiomycota (Wang et al. 2014), a phylogenetic group that diverged from Ascomycota more than 500 million years ago (Chang et al. 2021); *Malassezia* genus is composed of a group of dimorphic yeast dependent on lipids (Wu et al. 2015), which inhabit the skin surface of humans and a wide range of warm-blooded animals (Gueho et al. 1996) and constitute the predominant component of the cutaneous mycobiome in humans (Findley et al. 2013). Species in this clade typically have a commensal lifestyle, although they are also involved in the development of dermatological diseases (DeAngelis et al. 2005), and their possible mutualistic mechanism has been described (Li et al. 2018). Despite the difficulties involved in growing *Malassezia* strains under laboratory conditions, their presence has been detected, via molecular methods, in diverse habitats, such as deep-sea ecosystems (Le Calvez et al. 2009), Antarctic soils (Fell et al. 2006), corals, sponges, and cone snails (Amend 2014). This highlights the great ecological diversity and adaptability of *Malassezia*.

Unlike the Ustilaginomycetes, one of the phylogenetically related and extensively studied group of filamentous fungi (Wang et al. 2014), this clade contains significantly smaller genomes ranging from 7—9 Mb, with genes distributed across a variable number of chromosomes ranging from 6 to 9 (Boekhout et al. 1998; Sankaranarayanan et al. 2020). Recently, the public release of genome sequences from various *Malassezia* species has provided a valuable opportunity to widen the exploration of structure, function, and evolutive relation of telomerase catalytic and RNA subunits among Basidiomycota group of fungi; moreover, the differences in the life cycle, genome organization, and genetic content make them also attractive to compare whether the telomerase pathway for genome maintenance shares similarities in these 2 dimorphic pathogens; the findings suggest some analogies with telomerase RNA gene structure from *Ustilago maydis*.

## Materials and methods

### Identification of the putative TER loci in *Malassezia* species

The identification of the putative TER loci was performed following a previously described method (Chakrabarti et al. 2007). A comprehensive search was conducted across the genome of *Malassezia globosa* CBS7966 (GenBank accession: GCA_010232095.1) to identify regions containing at least one and a half copies of the expected template sequence (5′-CACTAA-3′) in its 6 possible permutations: 5′-CACTAACAC-3′, 5′-ACACTAACA-3′, 5′-AACACTAAC-3′, 5′-TAACACTAA-3′, 5′-CTAACACTA-3′, and 5′-ACTAACACT-3′. The candidate loci, along with short flanking sequences on each side, were subsequently used as search queries to identify orthologous sequences in the genomes of 18 additional *Malassezia* species (Supplementary Table 1) available to date via BLASTn (Altschul et al. 1990). Next, multiple sequence alignments were performed to identify conserved motifs within the candidate sequences to be the TER loci. The sequences obtained from the orthologous genes of the different *Malassezia* species were aligned via MAFFT (Katoh et al. 2002) with the G-INS-i strategy, and the –unalignlevel 0.8 and –leavegappyregion options. The alignment was visualized with BioEdit software (Hall T 1999), allowing for a comprehensive analysis of the conserved regions.

### Secondary structure prediction

Based on the built alignments, the total length of the potential genes was estimated by identifying the first and last well-conserved motifs within the explored intergenic regions. The first element corresponded to a conserved sequence with no similarity to known regulatory motifs, while the last element was identified as the potential Sm-binding site. Once the boundaries were established, the multiple sequence alignment was used as input to predict consensus secondary structures with RNAalifold (Bernhart et al. 2008). These structures were subsequently utilized as constraints in Mfold (Zuker 2003) to model the secondary structure of the *M. globosa* sequence. Additionally, the identification of the first stem of the pseudoknot was performed through a manual search following previously described criteria (Gunisova et al. 2009).

### Transcriptomic profiling of the putative *MgTER* gene

To obtain insights into the transcriptional activity of the putative *MgTER* gene, RNA-seq data available for *M. globosa* were downloaded (accession numbers: SRR2072129 to SRR2072131), and the quality of the raw paired reads was assessed via FastQC v0.11.9 (Andrews 2010). The removal of ribosomal RNA reads was subsequently performed with SortMeRNA software v4.2.0 (Kopylova et al. 2012). The trimming and removal of low-quality reads were performed with Trimmomatic v0.39 (Bolger et al. 2014), and reads with scores greater than 30 and a minimum length of 25 nt were retained for further analysis. Next, *de novo* assembly was performed with Trinity v2.15.1 (Grabherr et al. 2011) on the processed reads with the following parameters: –min_kmer_cov = 2 –min_contig_length = 100 –jaccard_clip –SS_lib_type FR. The reconstructed transcripts and processed reads were mapped to the reference genome with GMAP v23-02-17 (Wu and Watanabe 2005) and Bowtie 2 v2.4.4 (Langmead and Salzberg 2012), respectively. The alignments were visualized and explored with Integrative Genomics Viewer software v2.16.0 (Robinson et al. 2011), enabling a comprehensive examination of the putative gene and its genomic surroundings.

### Phylogenetic tree construction

A phylogenetic tree was constructed using the putative TER genes sequences from the 19 *Malassezia* species. The sequences were analyzed with MEGA11 software v11.0.13 (Tamura et al. 2021), employing the maximum likelihood approach with the HKY nucleotide substitution model. To assess the robustness of the tree groupings, 500 bootstrap replicates were performed.

## Results

### Identification of the putative *MgTER* gene

**Fig. 1.**
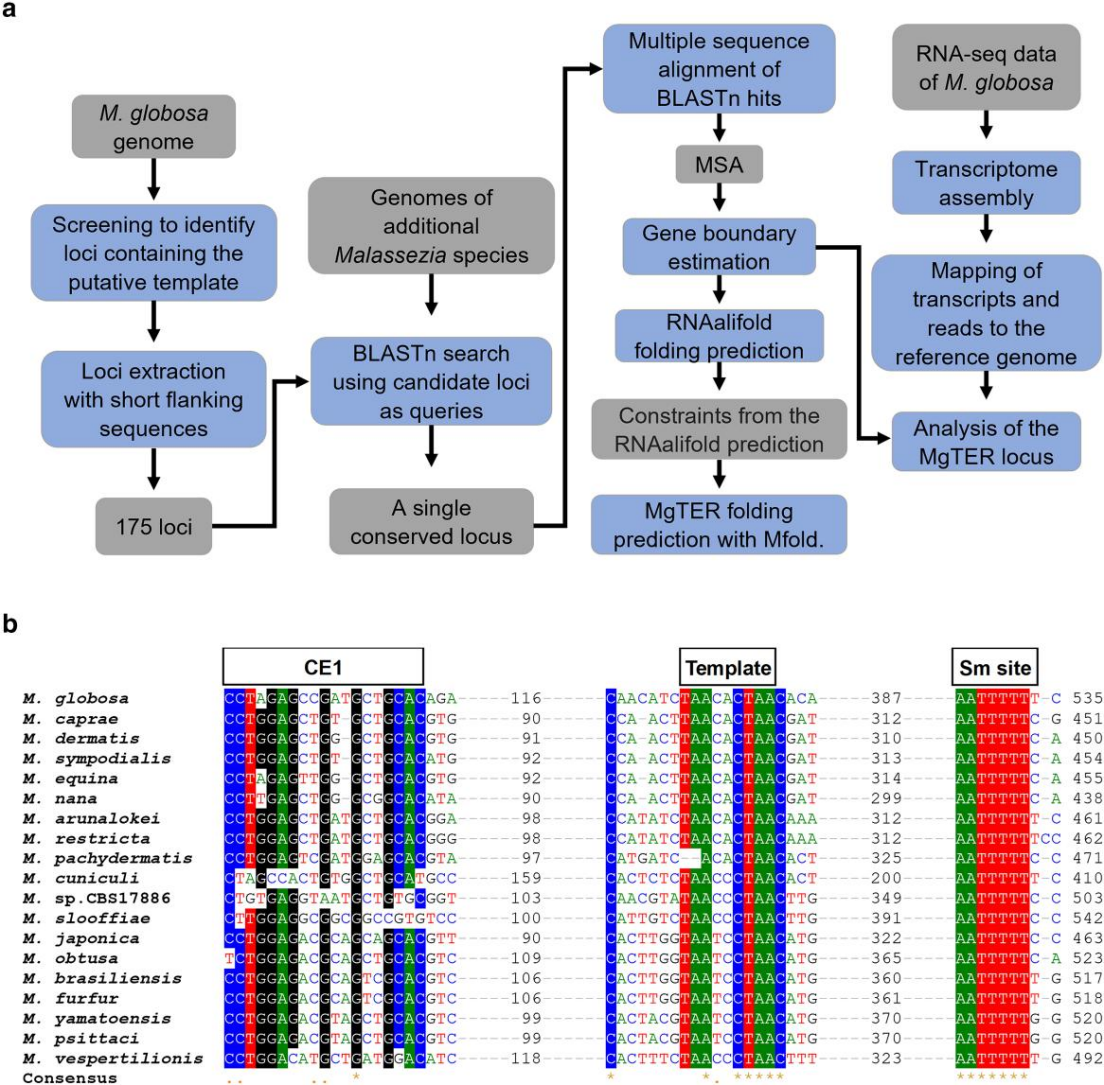
Approach for identifying putative TER loci in *Malassezia* species. a) Strategy for identifying candidate loci. Gray boxes represent input data or obtained results, while blue boxes indicate processes. The workflow begins with screening the *M. globosa* genome for loci containing the putative template sequence, followed by the extraction of loci with flanking sequences. These loci were used as queries in a BLASTn search against the genomes of 18 additional *Malassezia* species, identifying a single conserved locus across all species. A subsequent multiple sequence alignment of the hits was performed, and gene boundaries were estimated based on conserved motifs at the most distal ends. Constraints from RNAalifold predictions were applied for the folding prediction of MgTER using Mfold. Additionally, transcripts and reads were mapped to the reference genome to gain insight into MgTER locus. b) Multiple sequence alignment of the putative TER loci in *Malassezia* clade species. Within the candidate locus of the *MgTER* gene, 2 conserved motifs flank the intergenic region that contains the template domain. A conserved element (CE1) of unknown function was detected upstream of the template domain. Toward the 3′ end of the intergenic region, a highly conserved motif resembling a Sm-binding site was identified, marking the likely the start and end of the transcript. Each nucleotide is represented with a specific color (A: green, G: black, T: red, C: blue), and nucleotides conserved in at least 75% of the species are highlighted with a colored background. Boxed annotations indicate structural domains.

**Fig. 2.**
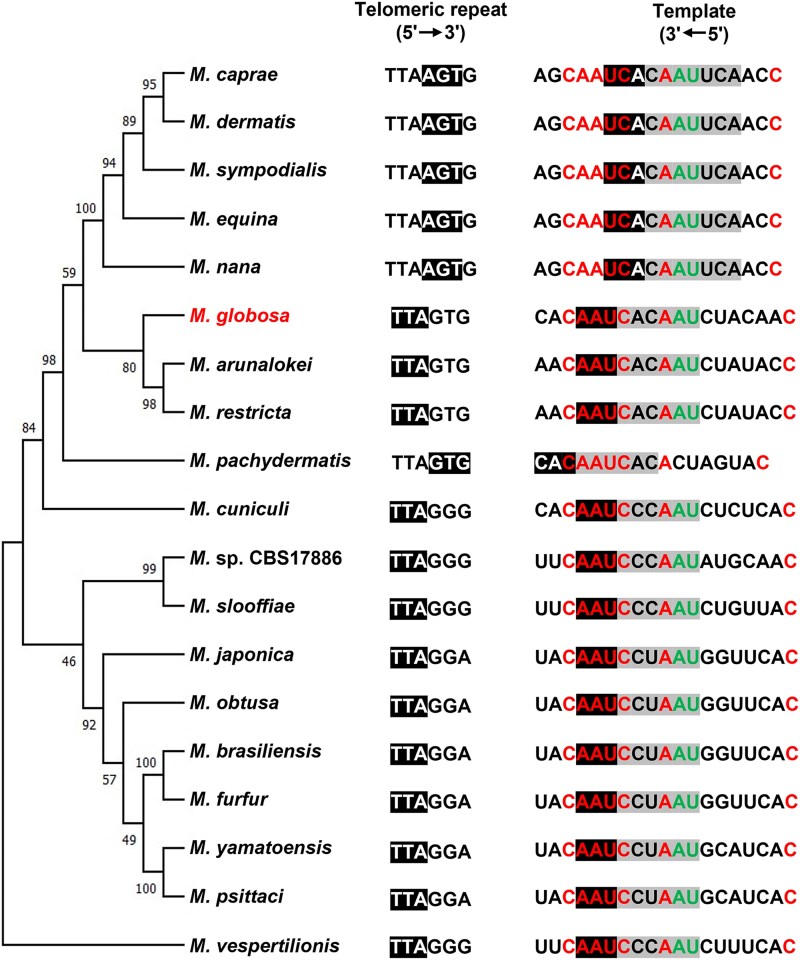
Telomeric repeats and phylogenetic tree of the putative TER gene in *malassezia* clade species. Evolutionary relationships of the TER gene among *Malassezia* species, telomeric repeats, and sequences containing the template domain (Columns 1 and 2, respectively) are shown. Nucleotides conserved in all species are shown in red, while nucleotides conserved in all species except *M. pachydermatis* are shown in green. The alignment region is highlighted in black, and the template region for retrotranscription is highlighted in gray. Point mutations within the region cause sliding of the template sequence and the generation of a new telomeric repeat.

The mining of TER template-domain sequences within the *M. globosa* genome and the search for orthologous regions allowed the identification of a unique intergenic region that was moderately conserved in all the examined *Malassezia* species. In all of them, this region was flanked at its 3′ end by a reading frame encoding a protein of unknown function that contained the conserved DUF1115 domain. At the 5′ end, the *NDK1* gene was found, which encodes a nucleoside diphosphate kinase; however, in at least 5 species a reading frame encoding a cysteine hydrolase was identified interspersed between *NDK1* and the TER loci (Supplementary Fig. 1). Once the orthologous regions of the MgTER locus from *M. globosa* were identified, the sequences were aligned and examined to identify conserved motifs (Supplementary Fig. 2). At first, the conserved motif of the putative template domain was examined, as shown in Fig. 1, and point mutations were identified and registered. These mutational changes within the expected template sequence matched in all 18 strains with the telomeric repeat previously reported for each species (Coelho et al. 2023) (Fig. 2).

Toward the 5′ end of template domain (TD), the first conserved region, termed conserved element 1 (CE1), was identified at an average distance of 112 nucleotides upstream of TD (Fig. 1). Subsequently, a search for potential promoter elements located upstream of the template domain, including the CE1 motif, was conducted. These sequences were analyzed using the TRANSFAC database (Matys et al. 2006) without results suggesting the presence of promoter elements in the analyzed regions. Aimed to predict whether the precursor transcript could undergo alternative splicing events to produce the mature transcript as occurs in *U. maydis* (Logeswaran et al. 2022), splicing-related motifs were searched in the 5′ region of the proposed precursor transcript. Similarly, the 3′ ends were analyzed for spliceosomal cleavage motifs resembling those reported at the 3′ end of telomerase RNA, which are associated with cleavage reactions in some yeasts and filamentous fungi (Box et al. 2008; Gunisova et al. 2009; Kuprys et al. 2013). None of these analyses yielded evidence supporting such hypotheses.

Toward the 3′ end, the consensus sequence 5′-AAUUUUU-3′ was identified, which is identical to the putative Sm-binding site of Ustilaginales (Logeswaran et al. 2022; Sanpedro-Luna et al. 2023). Sm-binding sites in numerous yeast species are close to the 3′ ends of telomerase RNA subunits, where they play essential roles ensuring proper RNA processing and stability (Gunisova et al. 2009; Leonardi et al. 2008; Seto et al. 1999). These sequences, therefore, signal the approximate termination point of the mature transcript. Based on the identification of the conserved Sm-binding site, this sequence was designated the 3′ boundary of the gene. In contrast, CE1, which is the most distal conserved element toward the 5′ end and lacks similarity to known regulatory elements, was initially hypothesized to be part of the mature transcript and was therefore designated the tentative 5′ boundary. This delineation yielded an estimated transcript length of 483 nucleotides. In early RNA secondary structure predictions, the transcript model included CE1 owing to its conservation and position at the 5′ end of the locus.

### The putative *MgTER* gene contains conserved hallmarks of telomerase RNA

**Fig. 3.**
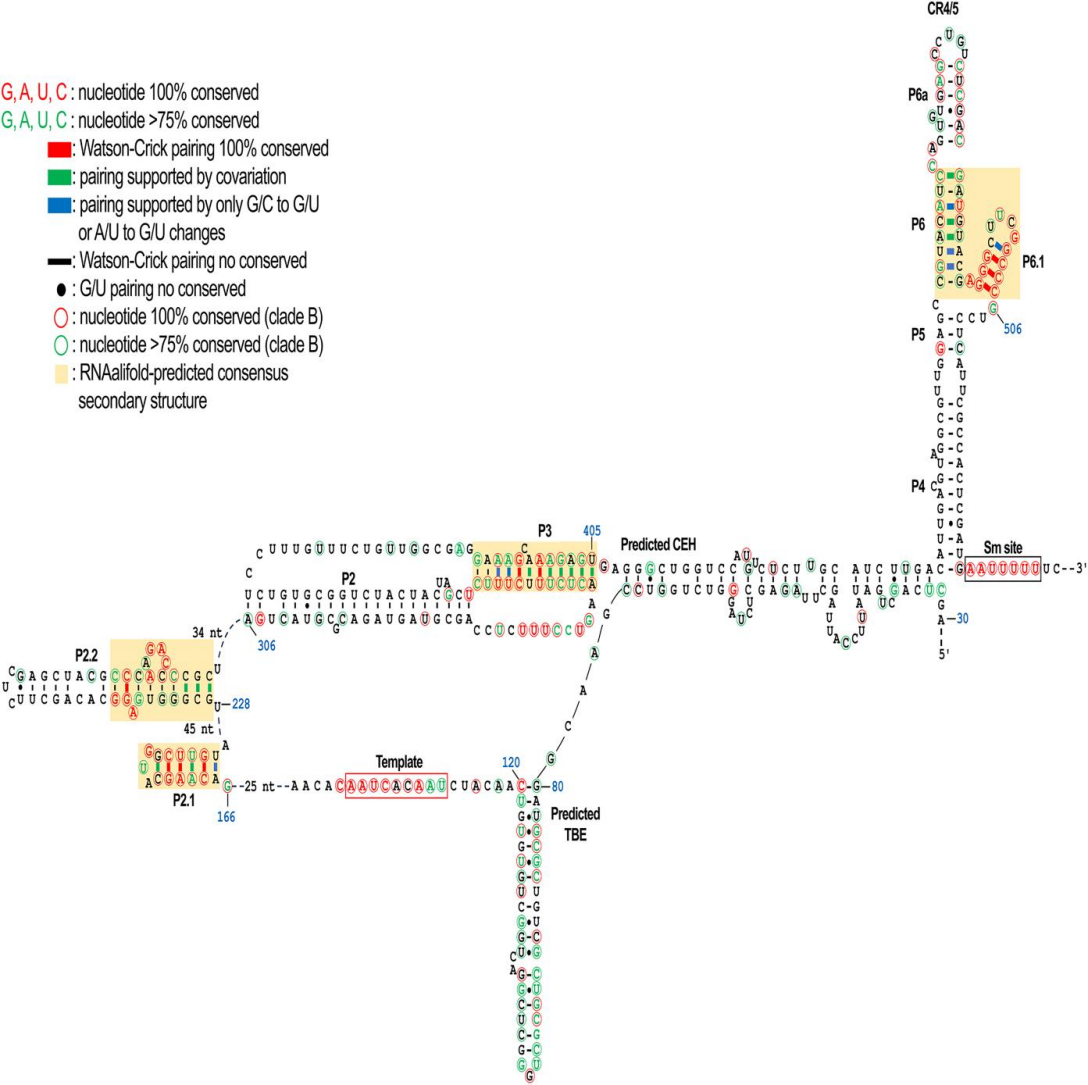
Proposed secondary structure of the TER subunit in *M. globosa*. The mature MgTER subunit in *M. globosa* has a length of around 500 nt and can fold into a secondary structure that contains the structural domains required for subunit activity, including the template domain, template boundary element (TBE), pseudoknot, CR4/5 domain, and Sm-binding site. The conserved nucleotides in all the examined species are displayed in red. Nucleotides conserved in at least 75% of the species are depicted in green. Highlighted regions in beige represent the predicted secondary structures supported by phylogenetic analysis. Watson–Crick base pairs that are 100% conserved are shown in red boxes, while base pairs supported by covariation are represented in green boxes. Blue boxes indicate pairing supported by limited nucleotide variations. Black dashes represent non-conserved Watson-Crick base pairs, and filled black circles indicate non-conserved G/U base pairs. Circles outlined in red indicate nucleotides that are fully conserved among clade B species, while those outlined in green represent nucleotides conserved in at least 75% of clade B species. Regions for which the secondary structure is not determined are shown using dashed lines, with the number of excluded residues indicated.

Once the transcript lengths were estimated, the aligned sequences were analyzed using RNAalifold, and at least 4 phylogenetically supported helices were predicted. These helices were subsequently used as constraints in Mfold to predict the secondary structure of MgTER. The obtained model resembles the telomerase RNA structures found in vertebrates and fungi (Chen et al. 2000; Qi et al. 2013); this includes structural elements analogous to those required for the biogenesis and activity of the mature transcript in other organisms. To refine the estimation of the 5′ boundary, a comparative approach was employed using the more compact sequence of *Malassezia cuniculi*, which presents fewer insertions between the conserved helices identified by RNAalifold. Mfold analyses of the *M. cuniculi* sequence revealed the formation of an arm beginning at position +69, which connects the central catalytic core to the terminal stem and encompasses the predicted core-enclosing helix (CEH) (Supplementary Fig. 3). Thus, CE1 was excluded from the folding predictions, and the sequence in *M. globosa* that aligned with the region around position +69 in *M. cuniculi* was used to refine the 5′ boundary. This refinement led to an updated secondary structure prediction for MgTER, with an estimated 5′ limit at position +33. The predicted structure includes a template domain, which is unambiguously identified by its sequence, a stem adjacent to the 5′ end that could function as a template boundary element (TBE), a pseudoknot structure, a terminal stem-helix, and the putative Sm-binding site (Fig. 3).

Downstream of the template domain, a moderately conserved region was found that resembles the typical 3 consecutive segments of U-A·U triple bases that stabilizes a triple helix configuration in the pseudoknot domain (Theimer et al. 2005; Ulyanov et al. 2007). This region has the potential to transcribe sequences with high contents of U and A residues. From this region, the segment designated P3 forms a stem that corresponds to the predicted stem 2 of the pseudoknot domain, as it is formed by U-A base pairs interspersed with C-G pairings and is supported by sequence covariation. In addition, conserved residues of U and C are present in loop 1, suggesting the potential to form U-A·U triple bases. Additionally, based on nucleotide covariations, 2 conserved stems were identified between the template region and the pseudoknot, which we arbitrarily designated P2.1 and P2.2. Similarly, the CR4/5 domain near the 3′ end was identified based on covariation and displays a structure analogous to the P6.1 helix (Logeswaran et al. 2021; Qi et al. 2013). Moreover, at the 3′ end, the putative Sm-binding site (Box et al. 2008; Leonardi et al. 2008; Seto et al. 1999) contains a highly conserved nucleotide sequence that remains invariant across all the examined species (Supplementary Fig. 2).

Although the same conserved structural elements, such as P2.1, P2.2, P3, and P6.1, were also present in the predicted secondary structure model of *M. cuniculi*, these features were connected by noticeably shorter segments. In some cases, such as between P2.2 and P2, the connecting sequences appeared to be absent. Additionally, adjacent to the template domain at the 5′ end, a predicted TBE-like structure was identified. In contrast to the corresponding region in MgTER, the stem harboring the TBE in *M. cuniculi* is longer, forming a well-defined helix with stable base pairing. Although this region did not show clear covariation signals on the basis of the global alignment and showed only a moderate distribution of conserved nucleotides, a focused alignment restricted to clade B species (Supplementary Fig. 4), which includes *M. globosa* (Wu et al. 2015), revealed a greater degree of nucleotide conservation in this region, thus supporting its potential functional relevance. Although a plausible CEH was identified downstream of the P3 stem in MgTER, minimal sequence conservation was observed in this region, even when the alignment was used restricted to closely related species. This limited conservation, together with the markedly reduced size of both the arm linking the catalytic core to the terminal stem and the short arm immediately preceding the CR4/5 domain in *M. cuniculi*, suggests that the folding of these regions is highly variable and likely species-specific. This variability may reflect structural adaptations unique to each species.

Moreover, consistent with the rapid evolution of telomerase RNA described in other organisms (Chen et al. 2000; Zappulla and Cech 2004), the comparative analysis among *Malassezia* species reveals greater divergence in their TER sequences compared to other of their noncoding RNAs. Specifically, TER identity among species in this clade ranges from 0.34 to 0.64, whereas other noncoding RNAs such as 5S and U2 in the same species show higher conservation, with identities ranging from 0.81 to 1.0 (Supplementary Table 2). Furthermore, when comparing the divergence of *MgTER* within the *Malassezia* clade to that of *Umter* among *Ustilago* species, we observe that TER divergence in *Malassezia* is not as pronounced as in *Ustilago*, where *Umter* identity is even lower, ranging from 0.24 to 0.44 (Supplementary Table 3). In both comparisons, 5S and U2 maintain similar identity levels across clades.

### Transcriptional activity of the putative *MgTER* gene

To investigate the transcriptional activity of the intergenic region containing the putative *MgTER* gene, paired-end reads were used for *de novo* transcriptome assembly and subsequently mapped to the reference genome. After processing the raw data, a total of 47,746,223 high-quality paired-end reads were retained, resulting in the assembly of 21,968 unique transcripts with an average length of 893 nucleotides. Assembly statistics are provided in Supplementary Table 4, while Supplementary Table 5 presents comprehensive details on raw data processing and sequence alignment.

**Fig. 4.**
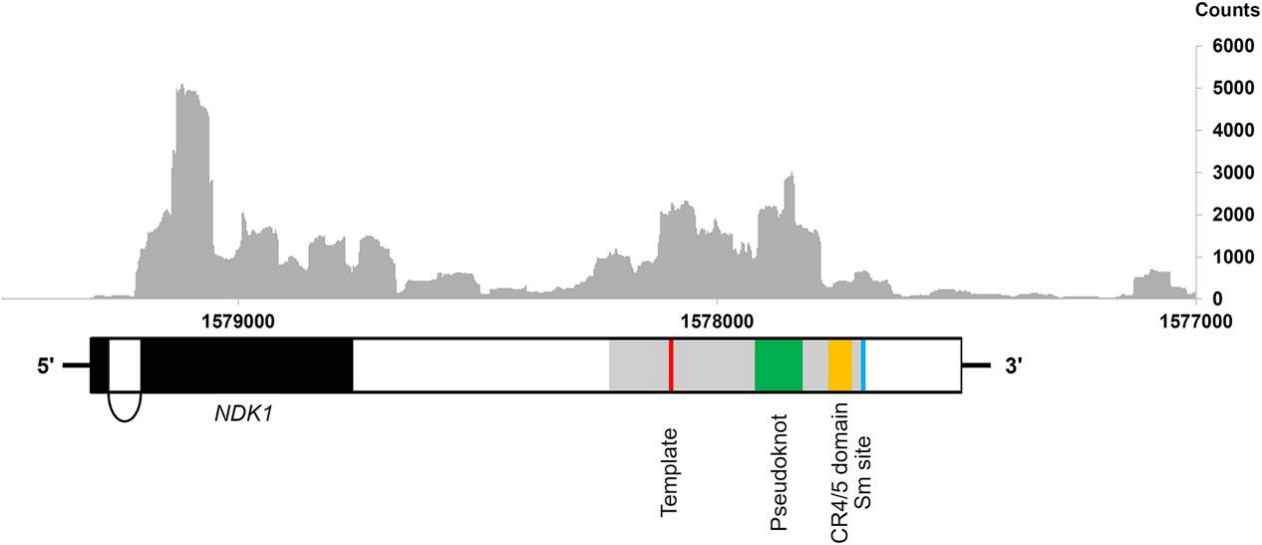
Representation of the putative *MgTER* gene identified on chromosome 1 of *M. globosa*. The locations of the structural domains within the putative *MgTER* gene (gray box) are represented by colored boxes. The upper part of the figure shows the density distribution of RNA-Seq reads along the gene. The results of the RNA-Seq read distribution confirmed the transcriptional capability of the intergenic region containing the putative *MgTER* gene, suggesting its potential to generate functional RNA in *M. globosa*.

Mapping of the assembled transcripts revealed a single long transcript (TRINITY_DN1050_c0_g2_i1, File S1) encompassing the entire sequence of the putative *MgTER* gene. This transcript, measuring 1,756 nt in length, contains an open reading frame at its 5′ end that encodes the ortholog of *NDK1*, a well-characterized nucleoside diphosphate kinase. The transcript comprises 2 exons, with a transcription start site located 25 nucleotides upstream of the *NDK1* start codon, and a long 3′ untranslated region (Fig. 4). It is noteworthy that the alignment of reads demonstrated active transcription across the entire intergenic region, with a notable increase in coverage over the sequence corresponding to the structural putative domains of TER.

Although libraries used for the transcriptome assembly of *M. globosa* in this work do not allow discerning between polyadenylated and nonpolyadenylated RNA populations from the transcribed region of TER, nor allow the precise discrimination between precursors and mature RNA transcripts, the results provide solid evidence of the transcriptional activity of the MgTER locus. Whereas the possibility that the observed transcript pattern in Fig. 4 could result from the overlap of 2 independent transcript populations due to the closeness of coding (*NDK1*) and non-coding (*MgTER*) genes in a narrow region should not be dismissed, it must also be considered the expression of a single transcript from *NDK1* promoter beyond Sm-binding site. Such bicistronic transcript could be congruent with the length of the uninterrupted reads abundance observed along the pair of genes; mature MgTER may result from post-transcriptional processing from the bicistronic transcript, presumably through endonucleolytic cleavages, in a manner analogous to the processing of the polycistronic transcript of *Um*Emi1 and *Um*TER (Logeswaran et al. 2022). Such endonucleolytic processing could account for the observed reduction in abundance at the 5′ and 3′ ends the predicted MgTER locus, as suggest the higher read coverage that aligns with the estimated mature transcript, while the capture of a series of post-transcriptional intermediates that eventually lead to the mature transcript could also contribute to the observed pattern. Further experiments are necessary to validate whether these proposed mechanisms indeed generate the mature MgTER transcript and to elucidate the biological implications of its overlap with *NDK1*.

## Discussion

### Bioinformatic identification of putative TER loci across the *Malassezia* clade

The availability of public databases and the increasing number of genomic sequences from Basidiomycota provide a valuable opportunity to continue studying TER in a phylogenetically diverse and understudied group of fungi. In this case, the bioinformatics approach, which takes advantage of the variability in known telomeric motifs among *Malassezia* species combined with a synteny-based strategy, enabled unambiguous identification of the loci harboring the putative TER gene within the *Malassezia* clade. The comparative study of the structure and sequence of TER between Basidiomycota and Ascomycota will help identify possible adaptations and evolutionary changes that have occurred in telomerase over time and in different fungal lineages.

While the identification of TER through conventional alignments of the gene encoding the catalytic subunit followed by immunoprecipitation has proven to be an effective technique (Leonardi et al. 2008; Webb and Zakian 2008), this approach is complex, highlighting that, despite its success, it has inherent challenges and limitations. On the other hand, although the computational approach simplifies identification, it is crucial to recognize that this strategy still has its own complexities and limitations. This approach is restricted to individual searches within narrow phylogenetic groups owing to the inherent plasticity of noncoding transcripts and the rapid evolution of genomes, as evidenced by the diversity of sequences that hinder reliable alignments and their identification in Saccharomycetes (Waldl et al. 2018).

### Structure and evolutionary divergence of TER in Basidiomycota

Interestingly, the putative TER gene identified in the *Malassezia* clade is similar in length to the TER gene in vertebrates (Chen et al. 2000), but unlike the latter, the presence of the putative Sm-binding site in this gene indicates that 3′ end processing follows the typical snRNA pathway of yeasts and some filamentous fungi (Egan and Collins 2012; Kuprys et al. 2013; Seto et al. 1999). On the other hand, the compact length of TER in the *Malassezia* clade contrasts sharply with the TER-encoding gene in Ustilaginales, which exhibits a more heterogeneous sequence and a greater length, ranging from 675 to 2,470 nt (Logeswaran et al. 2022; Sanpedro-Luna et al. 2023). This difference may be due to the fact that the *Malassezia* clade preserves a more ancestral form of TER, containing only the minimal elements required for telomerase complex assembly and activation, whose size is reminiscent of the designed Mini-T RNA version, which was engineered to remove sequences from the long RNA arms that are non-essential for core functionality, while preserving key protein-binding sites and structural elements critical for telomerase assembly and activity, maintaining telomerase activity *in vivo* and ensuring stable telomeres and cellular viability (Zappulla et al. 2005). Despite the lack of sequence identity, the similarity in size suggests that a compact RNA scaffold, whether naturally occurring or specifically designed, may be sufficient to preserve essential telomerase functions. This observation prompts intriguing questions regarding the functional and evolutionary significance of telomerase RNA compactness. Likewise, the reduced size of *Malassezia* TER genes may represent a product of evolutionary pressure to preserve a minimalistic structure, potentially driven by selective constraints that maintain the integrity of the sequence with minimal alterations, shaped by the genomic constraints inherent to the clade.

In contrast, the differences between *Malassezia* TER gene and *Um*TER gene, together TER sequences from species closely related to the latter, could be explained by dynamical DNA sequence changes resulting in addition and accumulation of sequences between essential motifs, likely generating dispensable regions, as occurred in TLC1 (Livengood et al. 2002; Zappulla et al. 2005). Additionally, the transcriptional fusion of *Um*TER gene with the UMAG_03168 locus, which encodes the *Um*Emi1 protein involved in meiosis (Logeswaran et al. 2022), could have contributed to the observed divergence. Notably, the homolog of *Um*Emi1 in *M. globosa* is located outside chromosome 1 (Supplementary Fig. 5), where *MgTER* is found. This positional disparity suggests that genomic rearrangements and differential transcriptional contexts may have influenced the evolutionary divergence of TER sequences between these species.

In addition, the reconstruction of a continuous transcript containing *NDK1* gene and predicted domains of TER, along with finding of divergence on the 5′ side of the intergenic sequence and the absence of conserved motifs outside the proposed TER loci, suggests that, as in the Ustilaginales, the mature TER transcript in the *Malassezia* clade also originates from a coding gene. Although limited information on TER in Basidiomycota is available to date, the conservation of a more compact gene linked to the ancestral form of TER suggests that TER-intron fusion at the 3′ end in the early Ascomycota lineages and the 3′ end processing mediated by the spliceosome (Qi et al. 2015) is exclusive to the phylum Ascomycota and occurred after the divergence from Basidiomycota.

### Diversity of telomeric motifs in *Malassezia*

The *Malassezia* clade contains 4 distinct telomeric repeats (Coelho et al. 2023) despite the overall conservation of TER gene length. This variation in telomeric motifs is attributable mainly to single-nucleotide substitutions within the template domain sequence. Additionally, alignment slippage caused by mutations in the regions flanking the template domain can also generate alternative sequences that encode the same telomeric motif, as observed in *Malassezia pachydermatis*. In other cases, this mechanism may underlie the emergence of distinct motifs, such as the TTAAGTG motif identified in certain species. Interestingly, species exhibiting the TTAAGTG motif still retain an intact template sequence capable of directing the synthesis of the TTAGTG motif. In these cases, it would be important to investigate whether the observed alignment and extension preferences are associated with sequence variations adjacent to the template domain and the formation of the TBE.

Furthermore, as shown by the phylogenetic analysis of the putative gene, the origin of these telomeric repeats is consistent with a common ancestor bearing the TTAGGG sequence (Fig. 2). This, in turn, is consistent with the idea that this sequence possibly corresponds to the most ancestral template sequence of TER across diverse lineages (Meyne et al. 1989; Podlevsky and Chen 2016). Simultaneously, the sustained utilization of templates exhibiting minor variations suggests the presence of discernible selective pressure, likely stemming from the imperative requirement for interaction with proteins associated with telomeres (Cervenak et al. 2019; Cervenak et al. 2017).

### Covariation signatures in the TER pseudoknot of *Malassezia*

Interestingly, in vertebrates and diverse fungal lineages, the nucleotide residues of the P3 stem and the uridine-rich loop that form the pseudoknot structure are strongly conserved (Chen et al. 2000; Lin et al. 2004; McMurdie et al. 2025; Tzfati et al. 2003). In this context, at least 3 consecutive U-residues are invariantly present in loop 1 across all examined *Malassezia* species, and there is also evidence of covariation, especially in the second half of the P3 stem. Both features could reflect a selective pressure to maintain crucial tertiary interactions for the formation and stability of the pseudoknot (Qiao and Cech 2008; Tzfati et al. 2003; Wang et al. 2016). Meanwhile, in species of clade B, widely conserved base pairs are observed in the P3 stem. However, it remains unclear whether the observed covariation reflects clade evolutionary distance or if it relates to coevolutionary events linked to sequence changes and alignment in the template domain.

Previous studies have shown that alterations in the sequence of stem 2 of the pseudoknot can induce template shifts (Tzfati et al. 2003). Furthermore, major plant clades exhibit covariation within the second half of the P3 while displaying different permutations of the same template sequence and divergence in TBE architecture (Song et al. 2019), suggesting a potential link between structural changes in the pseudoknot and the evolutionary dynamics of the template domain.

### Future directions

To assess whether the absence of the *MgTER* locus induces a senescent phenotype, future studies should evaluate the functional role of this locus in telomere maintenance via genetic manipulation using the mutagenesis systems that were successfully developed for *Malassezia* (Ianiri et al. 2019). Alternatively, a more conservative strategy involving RACE assays to map the transcript boundaries could provide sufficient information to refine the secondary structure model, which could then be validated through SHAPE assays (Wilkinson et al. 2006). Moreover, the identification of well-conserved motifs such as the P6.1 helix and the Sm-binding site within Ustilaginomycetes and Malasseziomycetes may facilitate the detection of TER homologs in more distantly related species using integrated approaches based on the construction of position-specific weight matrices and covariance models, as has been successfully applied in other lineages (Song et al. 2019; Xie et al. 2008).

## Conclusion

In this study, we successfully identified the putative gene encoding the RNA subunit of telomerase in 19 *Malassezia* species. The proposed TER gene in each of these species harbors the complementary sequence of the telomere repeat motif. As observed in orthologous genes from other yeasts and filamentous fungi, a putative Sm-binding site located at the 3′ end of this gene suggests its processing through the snRNA pathway. However, unlike similar genes in these other organisms, *MgTER* is more compact and homogeneous in length, with an estimated size similar to those of related genes in vertebrates. Additionally, it contains the necessary structural elements that support its functionality. The transcriptional activity of this gene was revealed by the analysis of RNA-seq data, reinforcing the idea of the biological role as TER for this putative gene.

Here we show how the implementation of bioinformatic analyses represents a highly efficient and agile alternative for obtaining biological insights and generating approaches that significantly simplify processes and guide the development of experimental strategies. The findings presented here strengthen the solid foundation of *in silico* research aimed at ultimately achieving experimental confirmation of gene function and activity, as we would expect in the context of telomere maintenance for *MgTER* and its potential involvement in other cellular processes. Ultimately, the study of telomerase regulation and evolution, which is based on information collected from diverse organisms, may contribute to a better understanding of the function and impact of telomerase components on cellular biology. In this context, the identification of the TER gene in *Malassezia* clade offers a unique opportunity to contrast the structural and organizational features of the telomerase ribonucleoprotein complex with those in other model organisms, providing insights into its evolutionary adaptations across diverse species.

## Data Availability

The genome and annotation data of *M. globosa* CBS7966, utilized as a reference, were obtained from NCBI (GCA_010232095.1 and GCA_000181695.2, respectively). The RNA-seq data of *M. globosa* used for the analysis were retrieved from NCBI (accession numbers: SRR2072129 to SRR2072131). The scripts and files utilized for both the assembly process and the corresponding statistical analyses can be accessed at https://github.com/Antonio-Sanpedro/Transcriptome_assembly. Detailed information on the reference sequences used for orthologous identification can be found in Supplementary Table 1. Additionally, the nucleotide sequence data of the putative TER genes reported are available in the Third Party Annotation Section of the DDBJ/ENA/GenBank databases under the accession numbers TPA: BK068774 to BK068792. Supplemental Material is available at figshare: https://doi.org/10.25387/g3.28555838.

## Supplemental Material

Supplemental material available at G3 online.
